# Impact of HTLV-1 infection on clinicopathological characteristics and tumour immune microenvironment in colorectal cancer

**DOI:** 10.1007/s00428-025-04074-w

**Published:** 2025-03-20

**Authors:** Rin Yamada, Kota Arima, Hiromu Yano, Yukio Fujiwara, Kohei Yamashita, Kosuke Kanemitsu, Norihisa Hanada, Jun-Ichirou Yasunaga, Masaaki Iwatsuki, Yoshiki Mikami, Yoshihiro Komohara

**Affiliations:** 1 Department of Cell Pathology, Graduate School of Medical Sciences, Kumamoto University, 1-1-1 Honjo, Chuo-Ku, Kumamoto, 860-8556 Japan; 2 Department of Diagnostic Pathology, Kumamoto University Hospital, Kumamoto, Japan; 3 Department of Surgery, Izumi General Medical Center, Kagoshima, Japan; 4 Department of Gastroenterological Surgery, Graduate School of Medical Sciences, Kumamoto University, Kumamoto, Japan; 5 Department of Hematology, Rheumatology and Infectious Diseases, Kumamoto University Hospital, Kumamoto, Japan

**Keywords:** Colorectal cancer, Tumour immune microenvironment, Human T-cell leukaemia virus type-1, Regulatory T cell, Forkhead box P3

## Abstract

Recent advances in anti-cancer therapy have indicated the significance of the tumour immune microenvironment in tumour progression and resistance to anti-cancer therapy. This study investigated primary colorectal cancer (CRC) tissues resected from 180 cases in a single institute in a region highly endemic for human T-cell leukaemia virus type 1 (HTLV-1) carriers. Among those 180 cases, 35 HTLV-1 carriers were identified. CRC patients who were HTLV-1 carriers were significantly older (mean age: 76.9 vs. 72.7 years, *P* = 0.0341), with a lower incidence of lymph node metastases (pN0: 91% vs. 65%, *P* = 0.0085), and lower tumour stages (stage III or IV: 11% vs. 36%, *P* = 0.0117) compared to non-carriers. HTLV-1 carriers tended to show a lower incidence of relapse, although the difference was not significant (*P* = 0.2272). The density of forkhead box P3-positive regulatory T cells (Tregs) was significantly higher in HTLV-1 carriers (median density: 132 vs. 89 cells/mm^2^, *P* = 0.0051). In situ hybridisation showed cells positive for *HTLV-1 basic leucine zipper factor*, likely representing lymphocytes located in stroma around the cancer nest. Our findings indicate that lymph node metastasis was significantly suppressed in CRC patients infected with HTLV-1. Since HTLV-1 infection reportedly impairs the immunosuppressive functions of Tregs, anti-cancer immune responses are potentially enhanced in CRC patients who are HTLV-1 carriers.

**Supplementary Information:**

The online version contains supplementary material available at 10.1007/s00428-025-04074-w.

## Introduction

Human T-cell leukaemia virus type 1 (HTLV-1) was the first retrovirus discovered in humans, identified in 1980 [1]. HTLV-1 is transmitted by cell-to-cell contact through breastfeeding, sexual intercourse, and blood [2–5]. The estimated number of HTLV-1 carriers worldwide has been estimated as 5–10 million, with several highly endemic regions including southwestern Japan, sub-Saharan Africa, South America, the Caribbean, and specific areas in the Middle East and Australo-Melanesia [6]. Among these, Japan is considered to have the largest number of HTLV-1 carriers in the world, estimated at approximately 1 million [6, 7]. HTLV-1 can lead to adult T-cell leukaemia/lymphoma defined as neoplastic proliferation of HTLV-1-infected T cells after a long latency period but also causes several non-neoplastic inflammatory conditions, including HTLV-1-associated myelopathy (HAM) and HTLV-1-associated uveitis (HAU) [8–10]. HTLV-1 is mainly detected in CD4-, CD25-, and forkhead box P3 (Foxp3)-positive T cells [11–13]. Foxp3 is a master transcription factor of regulatory T cells (Tregs), which constitutively express cytotoxic T lymphocyte antigen 4 (CTLA-4) and a high-affinity interleukin (IL)-2 receptor that includes CD25 [14–16]. CTLA-4 downregulates CD80 and CD86 on antigen-presenting cells, inhibiting T-cell activation, while the high-affinity IL-2 receptor consumes IL-2, leading to cytokine deprivation-induced apoptosis of effector T cells [15, 16]. Through these mechanisms, Tregs are generally considered to contribute to immune suppression. Recent studies have suggested that the immunosuppressive function of Tregs is impaired by HTLV-1 infection, triggering an inflammatory response and leading to inflammatory conditions such as HAM [8, 9].

On the other hand, the tumour immune microenvironment (TIME) has been highlighted alongside advances in immunotherapy for various cancers. In colorectal cancer (CRC), various immune cells, including Tregs, have been shown to influence patient survival and are thought to impact responses to immune checkpoint inhibitors [17]. HTLV-1 infection reportedly does not increase the risk of solid cancer [18]. However, no previous studies appear to have investigated associations between HTLV-1 infection and the TIME of solid cancers. The aim of this study was to examine the impact of HTLV-1 infection on clinicopathological characteristics and the TIME in patients with CRC.

## Materials and methods

### Patients

The study design was approved by the institutional review board of Kumamoto University (approval no. #2224) and Izumi General Medical Center (approval no. #51) in accordance with the guidelines for Good Clinical Practice and the Declaration of Helsinki. Participants comprised 191 consecutive CRC patients who underwent surgical resection at Izumi General Medical Center between 2012 and 2022. HTLV-1 infection was determined based on the presence of serum anti-HTLV-1 antibodies. Clinicopathological data were obtained from clinical records and pathological reports. Six patients with pTis or pT1a, three patients for whom tissue samples were unavailable, one patient who underwent pre-operative neoadjuvant chemotherapy, and one patient for whom the anti-HTLV-1 antibody status was not examined were excluded from analysis, leaving a total of 180 patients.

### Samples

Tissue samples were fixed with 10% neutral-buffered formalin and embedded in paraffin. For patients with double primary CRCs, the more advanced lesion was selected. For each of the 180 CRCs selected, two pathologists (R.Y. and Y.K.) who were blinded to information about the samples reviewed haematoxylin- and eosin-stained sections of all tissue specimens, and selected two representative areas containing the highest density of lymphocytes at the invasive front, considering tumour heterogeneity. TMA blocks were then constructed from the invasive front using a manual tissue microarrayer with a 5-mm-diameter core.

### Immunohistochemistry

Sections (thickness, 3 µm) obtained from TMA blocks were immersed in EDTA solution (pH 8.0) and heated in a pressure cooker for antigen retrieval. Mouse monoclonal antibodies against CD8 (clone C8/144B; Nichirei, Tokyo, Japan), CD163 (clone 10D6; Novocastra, Newcastle, UK), Foxp3 (clone 236A/E7; Abcam, MA, USA), mutL homolog 1 (MLH1) (clone ES05; Dako, Glostrup, Denmark), and mutS homolog 2 (MSH2) (clone FE11; Dako), rabbit monoclonal antibodies against CD3 (clone SP7; Nichirei), Iba1 (polyclonal; Wako, Tokyo, Japan), mutS homolog 6 (MSH6) (clone EP49; Dako), and postmeiotic segregation increased 2 (PMS2) (EP51; Dako) were used as primary antibodies. After reaction with the primary antibodies, sections were incubated with horseradish peroxidase-labelled secondary anti-mouse or anti-rabbit antibody (Nichirei). Immunoreactions were visualised using the diaminobenzidine system (Nichirei). Stained slides were digitally scanned with a Nanozoomer S20 scanner (Hamamatsu Photonics, Shizuoka, Japan). Cell counting of CD3-, CD8-, Foxp3-, Iba1, or CD163-positive cells in the tumour area was performed using HALO version 3.6.4134 (Indica Labs, Albuquerque, NM, USA), and the average density (in cells per square millimetre) of two cores per patient was calculated. Tumour area was defined as extending up to 500 µm beyond the tumour outline, without an attempt to evaluate different tumour compartments separately, such as tumour nests and stroma. Intact expression of all mismatch repair (MMR) proteins including MLH1, MSH2, MSH6, and PMS2 was defined as MMR-proficient (pMMR), while the complete loss of any MMR protein expression was determined as MMR-deficient (dMMR).

### Multiplex immunohistochemistry

Mouse monoclonal antibodies against CD4 (clone 1F6; Nichirei) and Foxp3, and rabbit monoclonal antibodies against CD3 were used for multiplex immunohistochemistry. Immunoreactions were visualised using aminoethyl carbazole substrate solution (Nichirei), and the process up to that point followed the same procedure described above. Stained slides were digitally scanned with the Nanozoomer S20 scanner. After scanning, destaining and antibody stripping were performed as previously described [19]. The section was then restained and the process of scanning, destaining, and antibody stripping was repeated. Colour deconvolution into pseudo-fluorescent images and the fusion of each image were performed using HALO to generate multichannel pseudo-fluorescent images.

### Chromogenic RNAin situhybridisation

Chromogenic RNA in situ hybridisation (ISH) was performed using an RNAscope 2.5 HD Duplex Detection Kit (Advanced Cell Diagnostics, Newark, CA, USA) according to the instructions from the manufacturer. A commercially available probe targeting *HTLV-1 basic leucine zipper factor* (*HBZ*) (Advanced Cell Diagnostics) was used. The RNAscope 2.5 Duplex Positive Control Probe (Advanced Cell Diagnostics) and the RNAscope 2-Plex Negative Control Probe (Advanced Cell Diagnostics) were used as the positive and negative control probes, respectively.

### Statistical analysis

All analyses were performed using GraphPad Prism version 9.4.0 (GraphPad Software, San Diego, CA, USA). For differences in clinicopathological characteristics, the chi-square test or Fisher’s exact test was used for categorical variables, and Student’s *t*-test was used for numerical variables. For differences in the cell counting of immunohistochemically positive cells, the Mann–Whitney *U* test was used. The cumulative survival rate was compared using the log–rank test. Values of *P* < 0.05 were considered statistically significant.

## Results

### Clinicopathological characteristics of CRC patients with a comparison between HTLV-1 carriers and non-carriers

**Fig. 1:**
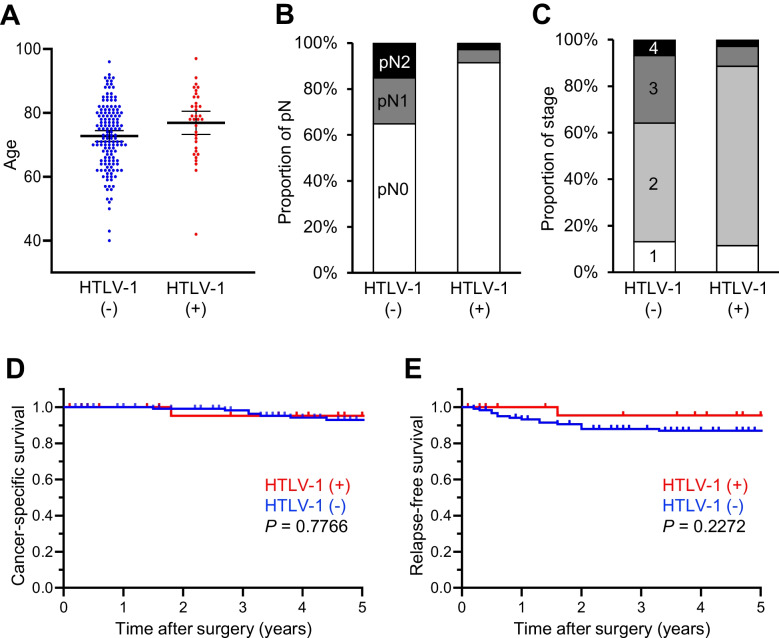
**A**–**C** Clinicopathological characteristics including age (**A**) and proportions of pN (**B**) and stage (**C**), showing significant differences between HTLV-1 carriers and non-carriers. Horizontal lines in **A** indicate means and 95% confidence intervals. **D**, **E** Kaplan–Meier survival curves. Panels compare cancer-specific survival (**D**) and relapse-free survival (**E**) between HTLV-1 carriers and non-carriers

The clinicopathological characteristics of patients are shown in Table 1. The 180 patients comprised 100 men (56%) and 80 women (44%), with a mean age of 73.5 years (range, 40–97 years). Seven patients (4%) had double primary cancers, for a total of 187 tumours. Among the 180 patients, 71 (39%), 104 (58%), and 5 (3%) had tumours on the right side (cecum to transverse colon), left side (descending colon to rectum), and bilaterally, respectively. Among the 187 tumours, 11 (6%), 39 (21%), 26 (14%), 17 (9%), 44 (24%), and 50 (27%) were located in the cecum, ascending colon, transverse colon, descending colon, sigmoid colon, and rectum, respectively. Most patients (82%) were graded as pT3 or higher. Regional lymph node metastases were detected in 54 patients (30%), while distant metastases were found in 11 patients (6%). Consequently, the CRC was classified as stage I in 23 patients (13%), stage II in 101 (56%), stage III in 45 (25%), and stage IV in 11 (6%). Only 7 tumours (4%) were poorly differentiated. Thirteen tumours (7%) showed dMMR. Among the 180 patients, 145 (81%) were negative for serum anti-HTLV-1 antibodies, while 35 (19%) were positive. HTLV-1 carriers were significantly older (mean age: 76.9 vs. 72.7 years, *P* = 0.0341), had a lower incidence of lymph node metastases (pN0: 91% vs. 65%, *P* = 0.0085), and consequently had CRCs at less advanced stages (stage III or IV: 11% vs. 36%, *P* = 0.0117) compared to non-carriers (Fig. 1A–C). No significant differences were observed in other clinicopathological characteristics. Subsequently, statistical analyses were reperformed only for pMMR cases, but no significant differences were observed (Supplementary Table 1).

**Table 1:** Clinicopathological characteristics of CRC patients with a comparison between HTLV-1 carriers and non-carriers

	Total (*n* = 180)	HTLV-1 (-) (*n* = 145)	HTLV-1 ( +) (*n* = 35)	*P* value
Mean age (range)	73.5 (40–97)	72.7 (40–96)	76.9 (42–97)	0.0341
Sex				0.8523
Man	100 (56%)	80 (55%)	20 (57%)	
Woman	80 (44%)	65 (45%)	15 (43%)	
Lesions in patients				0.1346
Single primary	173 (96%)	141 (97%)	32 (91%)	
Double primary	7 (4%)	4 (3%)	3 (9%)	
Tumor laterality				0.8455 (right vs. left)
Right side	71 (39%)	57 (39%)	14 (40%)	
Left side	104 (58%)	85 (59%)	19 (54%)	
Both sides	5 (3%)	3 (2%)	2 (6%)	
Tumor location				0.7988
Cecum	11 (6%)	9 (6%)	2 (5%)	
Ascending colon	39 (21%)	29 (19%)	10 (26%)	
Transverse colon	26 (14%)	22 (15%)	4 (11%)	
Descending colon	17 (9%)	14 (9%)	3 (8%)	
Sigmoid colon	44 (24%)	33 (22%)	11 (29%)	
Rectum	50 (27%)	42 (28%)	8 (21%)	
pT				0.2350 (pT1 or 2 vs. pT3 vs. pT4)
1	11 (6%)	10 (7%)	1 (3%)	
2	21 (12%)	17 (12%)	4 (11%)	
3	118 (66%)	91 (63%)	27 (77%)	
4	30 (17%)	27 (19%)	3 (9%)	
pN				0.0085
0	126 (70%)	94 (65%)	32 (91%)	
1	31 (17%)	29 (20%)	2 (6%)	
2	23 (13%)	22 (15%)	1 (3%)	
pM				0.6941
0	169 (94%)	135 (93%)	34 (97%)	
1	11 (6%)	10 (7%)	1 (3%)	
Stage				0.0117 (I vs. II vs. III or IV)
I	23 (13%)	19 (13%)	4 (11%)	
II	101 (56%)	74 (51%)	27 (77%)	
III	45 (25%)	42 (29%)	3 (9%)	
IV	11 (6%)	10 (7%)	1 (3%)	
Tumor differentiation				> 0.9999
Well/moderate	173 (96%)	139 (96%)	34 (97%)	
Poor	7 (4%)	6 (4%)	1 (3%)	
MMR status				0.7195
pMMR	167 (93%)	135 (93%)	32 (91%)	
dMMR	13 (7%)	10 (7%)	3 (9%)

Although cancer-specific and relapse-free survival rates did not differ significantly between HTLV-1 carriers and non-carriers (*P* = 0.7766 and 0.2272, respectively), HTLV-1 carriers tended to show a lower incidence of relapse (Fig. 1D, E).

### Measurement of immune cell densities in the TIME

**Fig. 2:**
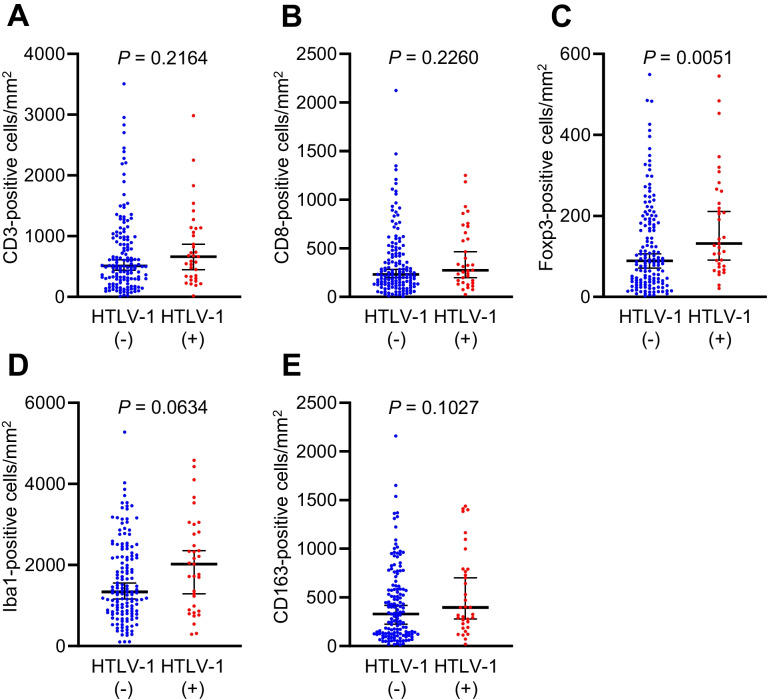
Immune cell densities in the tumour microenvironment between HTLV-1 carriers and non-carriers. Horizontal lines indicate medians and 95% confidence intervals. **A** CD3. **B** CD8. **C** Foxp3. **D** Iba1. **E** CD163

**Fig. 3:**
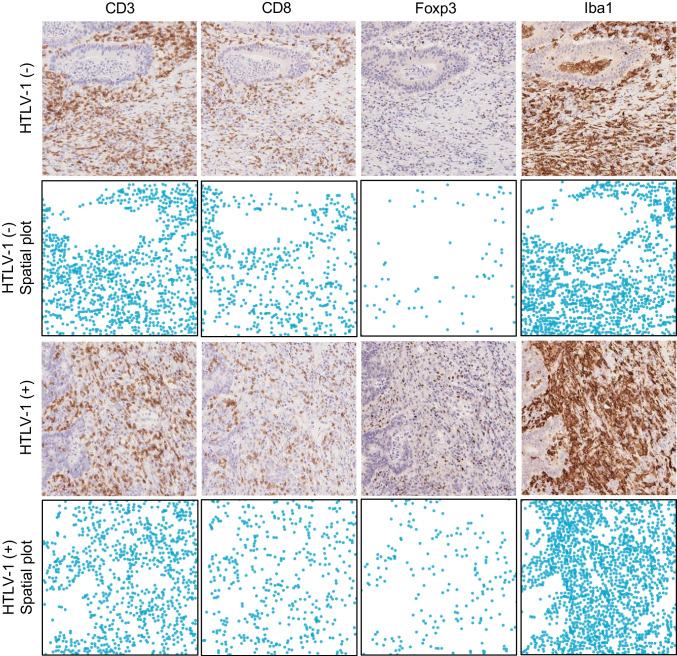
Densities of immune cells in representative cases. From top to bottom: non-carrier, spatial plot of non-carrier, HTLV-1 carrier, and spatial plot of HTLV-1 carrier. From left to right: CD3, CD8, Foxp3, and Iba1. The HTLV-1 carrier shows a higher density of Foxp3-positive cells compared to the non-carrier, while densities of CD3-, CD8-, and Iba1-positive cells show little difference

**Fig. 4:**
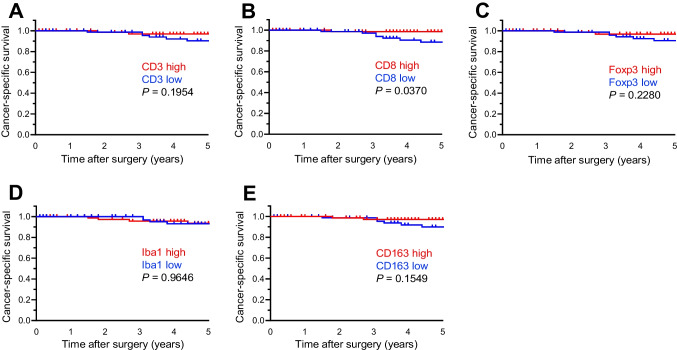
Kaplan–Meier survival curves. Panels compare cancer-specific survival between high- and low-immune-cell-density groups based on the median value. **A** CD3. **B** CD8. **C** Foxp3. **D** Iba1. **E** CD163

**Fig. 5:**
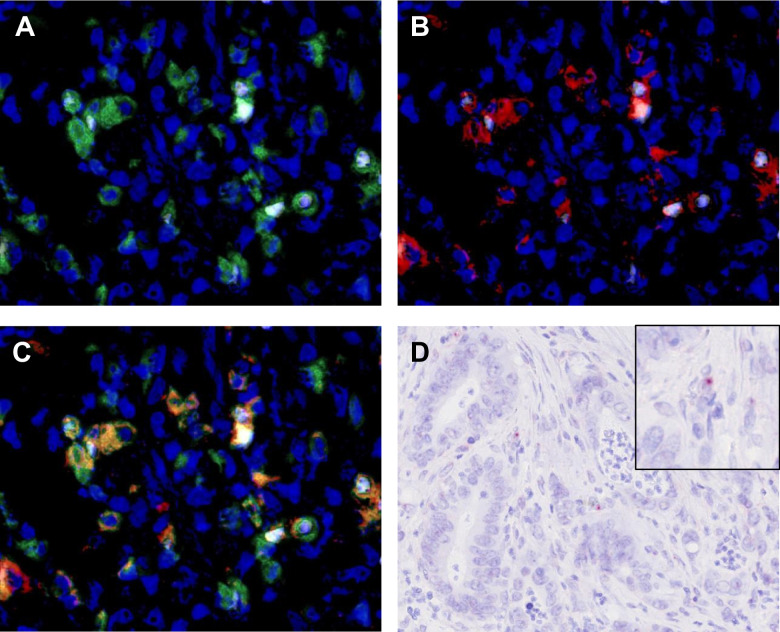
**A**–**C** Multichannel pseudo-fluorescent images (blue: DAPI; green: CD3; red: CD4; white: Foxp3). **A** Foxp3 and CD3. **B** Foxp3 and CD4. **C** Foxp3, CD3, and CD4. **D** Chromogenic RNA ISH. HBZ-ISH-positive cells, most likely representing lymphocytes, are detected around cancer cells. Inset shows a higher magnification

Immune cell densities in the TIME are shown in Fig. 2. The density of Foxp3-positive cells was significantly higher in HTLV-1 carriers (median density: 132 vs. 89 cells/mm^2^, *P* = 0.0051) compared to non-carriers. No significant differences were observed in densities of CD3- (median density: 614 vs. 510 cells/mm^2^, *P* = 0.2164), CD8- (median density: 277 vs. 233 cells/mm^2^, *P* = 0.2260), Iba1- (median density: 2021 vs. 1338 cells/mm^2^, *P* = 0.0634), or CD163-positive cells (median density: 397 vs. 330 cells/mm^2^, *P* = 0.1027) between HTLV-1 carriers and non-carriers. Representative cases are shown in Fig. 3. Furthermore, when cancer-specific survival was analysed by dividing patients into high- and low-immune-cell-density groups based on the median value, the CD8-high group exhibited longer cancer-specific survival (*P* = 0.0370) (Fig. 4A–E). The Foxp3-high group also exhibited a higher density of CD3-, CD8-, Iba1-, and CD163-positive cells (*P* < 0.0001) (Supplementary Fig. 1). Multichannel pseudo-fluorescent images are shown in Fig. 5A–C. Foxp3-positive cells were also CD3- and CD4-positive, confirming that these Foxp3-positive cells were indeed Tregs.

A portion of the mononuclear cells, likely lymphocytes surrounding the cancer cells, were positive for HBZ-ISH (Fig. 5D). As HBZ is a viral gene of HTLV-1, this finding indicated that HTLV-1 was present in the TIME [20].

## Discussion

In this study, we demonstrated that CRC patients who were HTLV-1 carriers were older, had a lower incidence of lymph node metastases, consequently had CRCs at less advanced stages, and tended to have a lower incidence of relapse compared to non-carriers. Additionally, we observed a higher density of Foxp3-positive Tregs in the TIME where HTLV-1 was present. Furthermore, a high density of CD8-positive cells was associated with longer cancer-specific survival.

HBZ leads to the upregulation of Foxp3 by enhancing transforming growth factor β signalling through interactions with mothers against decapentaplegic homolog 3/p300 [21]. The number of Tregs in peripheral blood is higher in asymptomatic HTLV-1 carriers and patients with HAM compared to non-carriers [22]. To the best of our knowledge, the present study is the first to investigate the TIME for solid cancers in HTLV-1 carriers.

Tregs suppress the induction of tumour antigen-specific effector T cells [23]. A high density of Tregs has been reported to correlate with worse clinical outcomes in several solid cancers, including breast cancer, gastric cancer, hepatocellular carcinoma, metastatic clear cell renal cell carcinoma, cervical squamous cell carcinoma, and lung adenocarcinoma [24–29]. In contrast, many reports have shown that high Treg density in CRC either correlated with favourable clinical outcomes or was not predictive [30–35]. The association between Treg density and regional lymph node status appears variable, with one study showing a positive correlation, but another showing a negative correlation, similar to our results [33, 34]. CD8-positive cytotoxic T cells are considered a major driver of anti-tumour immunity. A high density of CD8-positive cells has been reported to be associated with better clinical outcomes, consistent with our findings [30, 35, 36].

Although reasons for the differing associations between Treg density and clinical outcomes in CRC compared to non-CRC remain unclear, some authors have speculated that this may be due to differences in the composition of Treg fractions at the tissue site [37, 38]. Depending on the expression of CD45RA and Foxp3, Tregs can be subdivided into three fractions: CD45RA^+^FoxP3^low^ naive Tregs, CD45RA^−^FoxP3^high^ effector Tregs (eTregs), and CD45RA^−^FoxP3^low^ non-suppressive Tregs (non-Tregs) which secret proinflammatory cytokines such as IL-17, IL-2, and IFN (interferon)-γ [39, 40]. When CRCs were classified into two groups based on the estimated degree of non-Treg infiltration, the non-Treg-predominant group showed better survival than the eTreg-predominant group [41]. Further, in the eTreg-predominant group, high Foxp3 mRNA expression was associated with poor prognosis, whereas in the non-Treg-predominant group, high Foxp3 mRNA expression tended (but not significantly) to be associated with better prognosis [41]. This suggests that in CRC, not only the number of Tregs, but also the specific fraction of Tregs that is dominant in the tumour tissue is important for predicting prognosis, and that high non-Treg infiltration activates anti-tumour immunity.

Disordered suppressive function and the production of proinflammatory cytokines by Tregs have also been observed in HTLV-1 infection. In transgenic mice expressing HBZ, HBZ suppressed the expression of CTLA-4 and glucocorticoid-induced tumor necrosis factor receptor family-related protein (GITR), which are critical for Treg function, by physically interacting with Foxp3 and nuclear factor of activated T cells, and Tregs can produce IFN-γ [42, 43]. Suppression of CTLA-4 and GITR, as well as overproduction of IFN-γ, are also observed in the Tregs of HAM patients, and the increase in IFN-γ-producing Tregs contributes to the development of HAM [8, 9]. Such inflammatory conditions associated with HTLV-1 have also been observed in the intraocular fluid of patients with HAU [10]. Further, HTLV-1 has been detected in several organs, and has been suggested to be associated with various inflammatory diseases, including alveolitis/bronchiectasis, arthritis, and myositis [44–46]. In the TIME of CRC, HTLV-1 may similarly lead to impaired Treg suppression and increased inflammatory cytokine production, which could be associated with better prognostic factors, including a lower incidence of lymph node metastases, less advanced stage, and a tendency toward a lower incidence of relapse, as observed in this study.

This study showed a limitation in that examination of specific Treg fractions was not performed, as only formalin-fixed paraffin-embedded tissues were available, and immunohistochemistry is unable to distinguish between different Treg fractions [41].

In conclusion, this study is the first to examine the impact of HTLV-1 infection on clinicopathological characteristics and the TIME in solid cancer patients. CRC patients who were HTLV-1 carriers were older, had a lower incidence of lymph node metastases, showed less advanced CRC stage, and tended to have a lower incidence of relapse compared to non-carriers. A higher density of Tregs was observed in the TIME where HTLV-1 was present. As previously described, HTLV-1 may impair Treg suppression and increase inflammatory cytokine production in the TIME, which could be linked to these favourable prognostic factors.

## Supplementary Information

Below is the link to the electronic supplementary material.

ESM 1 (PPTX 266 KB)

ESM 2 (DOCX 33.3 KB)

## Data Availability

The datasets generated during and/or analysed during the current study are not publicly available due to the privacy of research participants or ethical restriction but are available from the corresponding author on reasonable request.
