# Supplementary material for: Impact of HTLV-1 infection on clinicopathological characteristics and tumour immune microenvironment in colorectal cancer
Source: Virchows Arch. 2025 Mar 20;487(4):853–63. doi: 10.1007/s00428-025-04074-w (PMC12546507; doi:10.1007/s00428-025-04074-w)
Supplement: Supplementary file 1 — (PPTX 266 KB) [file 428_2025_4074_MOESM1_ESM.pptx]

## Slide 1
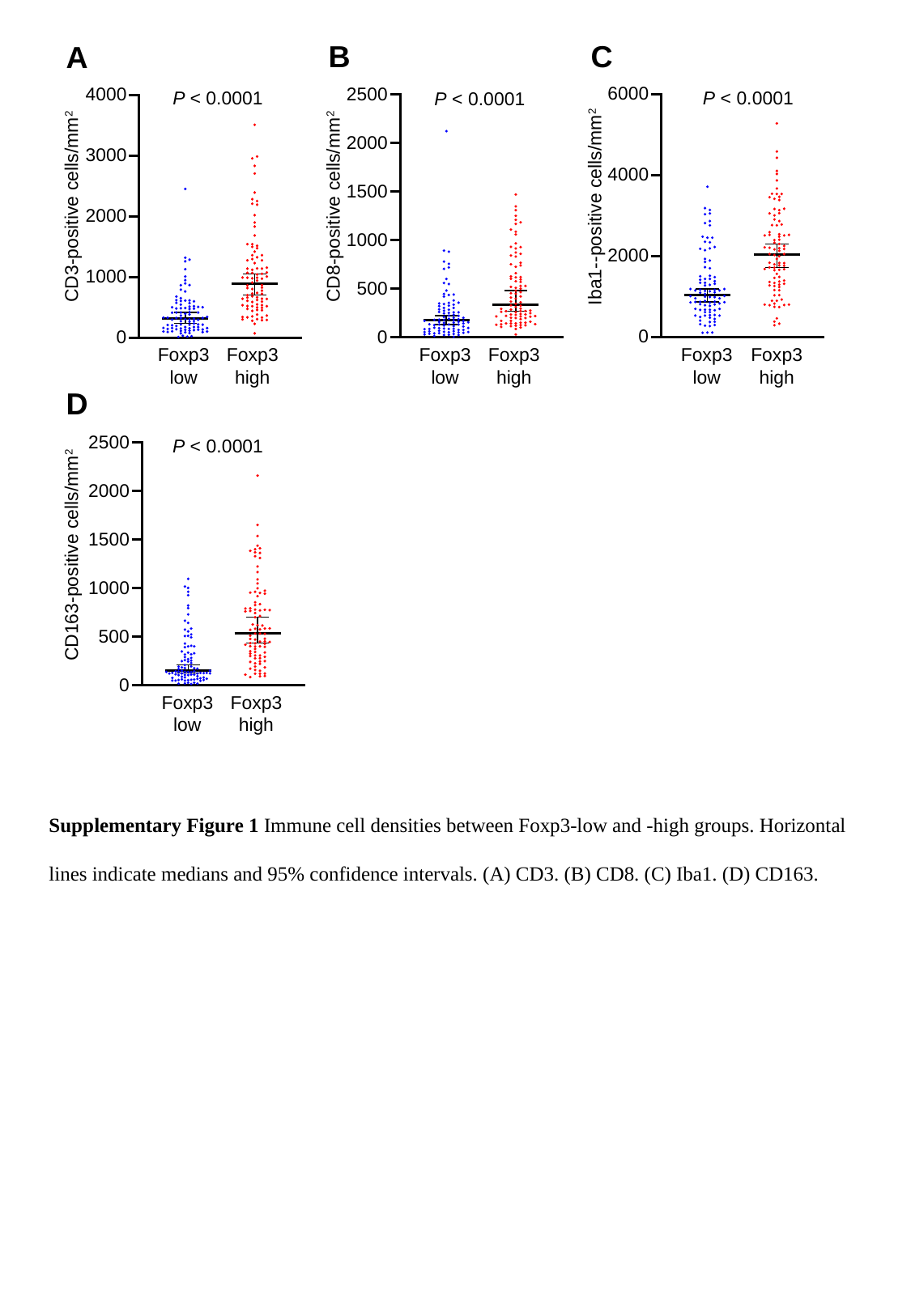

B
C
A
P < 0.0001
P < 0.0001
P < 0.0001
Iba1--positive cells/mm2
CD3-positive cells/mm2
CD8-positive cells/mm2
Foxp3
low
Foxp3
high
Foxp3
low
Foxp3
high
Foxp3
low
Foxp3
high
D
P < 0.0001
CD163-positive cells/mm2
Foxp3
low
Foxp3
high
Supplementary Figure 1 Immune cell densities between Foxp3-low and -high groups. Horizontal lines indicate medians and 95% confidence intervals. (A) CD3. (B) CD8. (C) Iba1. (D) CD163.
