# Supplementary material for: Impact of HTLV-1 infection on clinicopathological characteristics and tumour immune microenvironment in colorectal cancer
Source: Virchows Arch. 2025 Mar 20;487(4):853–63. doi: 10.1007/s00428-025-04074-w (PMC12546507; doi:10.1007/s00428-025-04074-w)
Supplement: Supplementary file 2 — (DOCX 33.3 KB) [file 428_2025_4074_MOESM2_ESM.docx]

| Supplementary Table 1 Clinicopathological characteristics of pMMR CRC patients with a comparison between HTLV-1 carriers and non-carriers | | | | |
| --- | --- | --- | --- | --- |
|  | Total (n = 167) | HTLV-1 (-) (n=135) | HTLV-1 (+) (n = 32) | *P* value |
| Mean age (range) | 72.9 (40–97) | 72.1 (40–96) | 76.2 (42–97) | 0.0450 |
|  |  |  |  |  |
| Sex |  |  |  | 0.8692 |
| Man | 97 (58%) | 78 (58%) | 19 (59%) |  |
| Woman | 70 (42%) | 57 (42%) | 13 (41%) |  |
|  |  |  |  |  |
| Lesions in patients |  |  |  | 0.1296 |
| Single primary | 160 (96%) | 131 (97%) | 29 (91%) |  |
| Double primary | 7 (4%) | 4 (3%) | 3 (9%) |  |
|  |  |  |  |  |
| Tumour laterality |  |  |  | > 0.9999 (right vs. left) |
| Right side | 60 (36%) | 49 (36%) | 11 (34%) |  |
| Left side | 102 (61%) | 83 (61%) | 19 (59%) |  |
| Both sides | 5 (3%) | 3 (2%) | 2 (6%) |  |
|  |  |  |  |  |
| pT |  |  |  | 0.1798 (pT1 or 2 vs. pT3 vs. pT4) |
| 1 | 10 (6%) | 9 (7%) | 1 (3%) |  |
| 2 | 20 (12%) | 16 (12%) | 4 (13%) |  |
| 3 | 110 (66%) | 85 (63%) | 25 (78%) |  |
| 4 | 27 (16%) | 25 (19%) | 2 (6%) |  |
|  |  |  |  |  |
| pN |  |  |  | 0.0050 |
| 0 | 117 (70%) | 87 (64%) | 30 (94%) |  |
| 1 | 28 (17%) | 27 (20%) | 1 (3%) |  |
| 2 | 22 (13%) | 21 (16%) | 1 (3%) |  |
|  |  |  |  |  |
| pM |  |  |  | 0.6890 |
| 0 | 157 (94%) | 126 (93%) | 31 (97%) |  |
| 1 | 10 (6%) | 9 (7%) | 1 (3%) |  |
|  |  |  |  |  |
| Stage |  |  |  | 0.0092 (I vs. II vs. III or IV) |
| I | 21 (13%) | 17 (13%) | 4 (13%) |  |
| II | 94 (56%) | 69 (51%) | 25 (78%) |  |
| III | 42 (25%) | 40 (30%) | 2 (6%) |  |
| IV | 10 (6%) | 9 (7%) | 1 (3%) |  |
|  |  |  |  |  |
| Tumour differentiation |  |  |  | > 0.9999 |
| Well/moderate | 164 (98%) | 132 (98%) | 32 (100%) |  |
| Poor | 3 (2%) | 3 (2%) | 0 (0%) |  |
